# Onset Time of Spinal Anaesthesia in Pregnant Females in Knee-Chest Position: A Randomized Controlled Study

**DOI:** 10.5152/TJAR.2021.919

**Published:** 2022-02-01

**Authors:** Purnima Narasimhan, Heena Garg, Souvik Maitra, Devalina Goswami, Shailendra Kumar, Neisevilie Nisa, Riddhi Kundu, Puneet Khanna

**Affiliations:** Department of Anaesthesiology, Pain Medicine and Critical Care, All India Institute of Medical Sciences, New Delhi, India

**Keywords:** Anaesthesia, cesarean section, knee-chest position, sensory block level, subarachnoid block

## Abstract

**Objective:**

To assess the efficacy of knee-chest position in shortening the time of spinal induction in pregnant women undergoing elective cesarean section. We also assessed for any untoward adverse events that might limit their usefulness in real-life clinical scenarios.

**Methods:**

Prospective, randomized controlled study was done in maternity operating room of tertiary care institution in 45 ASA II pregnant women undergoing elective cesarean section under spinal anaesthesia. Patients were randomly assigned to groups S (supine) and K (knee-chest position). After performing subarachnoid block (9 mg of 0.5% hyperbaric bupivacaine and 25 µg fentanyl) in the sitting position, women in group K were maintained in the knee-chest position for 60 seconds. Time to attain block height of T6 and maximum sensory blockade, intraoperative hemodynamics, Bromage score, intraoperative fluid, vasopressor requirement, and respiratory parameters were recorded. The newborn was evaluated using Apgar scores at 1 and 5 minutes.

**Results:**

Data of 45 patients were analyzed. Time to attain T6 block height (group K = 2.1 ± 0.65 minutes, 95% CI: 1.83-2.39; group S = 6.4 ± 0.77 minutes, 95% CI: 6.10-6.78) and time to achieve maximum sensory block height were significantly lower in group K (group K = 3.2 ± 1.35 minutes, 95% CI: 2.61-3.78; group S = 6.6 ± 0.89 min, CI: 6.19-6.98). The degree of motor block was higher in group K than that of group S at 2 minutes (*P* = .0002), 4 minutes (*P* < .0001), and 6 minutes (*P* < .0001), with no difference at 8 minutes. No statistically significant difference was observed in fluids and vasopressors requirement intraoperatively.

**Conclusions:**

This study provides evidence that the onset of adequate surgical anaesthesia for the cesarean section can be hastened by placing the patient in the knee-chest position for a minute after performing the subarachnoid block in the sitting position.

**Main Points**

1. Hastening the onset of spinal anaesthesia can be helpful in category 1 cesarean sections to avoid general anaesthesia.
2. Increasing the epidural pressure with knee-chest position can help spread of cerebrospinal fluid cranially leading to a rapid onset of action of spinal anaesthesia.
3. The current study provides enough evidence that the onset of adequate surgical anaesthesia for the cesarean section can be hastened by placing the patient in a knee-chest position for a minute immediately after the administration of subarachnoid block.
4. The hemodynamic effects of this technique were safe in our patient population.

## Introduction

A subarachnoid block is the most widely accepted technique of choice for pregnant women undergoing commonly elective cesarean section. It avoids the risks inherent to the performance of general anaesthesia and is generally safer for both the mother and the newborn.^1^ However, the time duration from the performance of a subarachnoid block to obtain adequate surgical block is often wide and variable.

Pregnant women with category 1 indication for cesarean section (immediate threat to the life of woman or fetus) often receive general anaesthesia, as it is a faster and more reliable option compared to the subarachnoid block.^2,3^

Few studies have attempted to find the time required for adequate surgical conditions following subarachnoid block. However, there are several other studies with a range of 4-12 minutes to T4 level in obstetric literature.^4,5^ Kathirgamanathan et al.^6^ found the median total time to readiness for surgery with a spinal anaesthetic was 8 minutes 52 seconds compared to 1 minute 56 seconds for general anaesthesia. To avoid this delay, Kinsella et al.^7^ proposed a technique of “rapid sequence spinal” anaesthesia, in which they used minimum sterile precautions and used a “no-touch” technique for the performance of the block. The authors reported that the median time from injection until the assessment of a satisfactory block was 4 minutes, and the total time to induce spinal anaesthesia to reach a sensory block level of T10 was 8 minutes in their study.^7^

Johnston et al.^8^ measured the pressure changes in the epidural space with changes in maternal position using an epidural catheter and connecting it to a pressure transducer. The authors noted that the epidural pressures were highest when the mother was supine with legs drawn up over the abdomen. They hypothesized that locally induced increase in epidural pressures can be transmitted to cerebrospinal fluid (CSF) in the lumbosacral region via engorgement of epidural veins, thereby creating a pressure gradient facilitating propulsion of CSF cranially.^8^

We hypothesized that increased epidural pressure from the knee-chest position (KCP) immediately after spinal anaesthesia will shorten the time to reach maximal block height. As our literature search did not yield any previous studies reporting the extent and hastening of surgical anaesthesia after KCP in pregnant women following subarachnoid block, this interventional study was done to assess the feasibility and effectiveness of this maneuver for elective cesarean section.

The primary outcome of this study was to compare spinal block onset times (measured from subarachnoid drug injection till attainment of T6 block height) between the KCP and supine positions in pregnant women. The secondary outcomes were to assess the incidence of hypotension and bradycardia, the maximum height of sensory blockade obtained, and fluid and vasopressor requirements between the 2 groups.

## Methods

This randomized controlled study was conducted in a tertiary care teaching hospital in India, after receiving Institutional Review Board approval. The study was registered in the National Clinical Trial Registry of India (www.ctri.nic.in, Reg No. CTRI/2016/05/006943). Written informed consent was obtained from 45 pregnant women scheduled for elective cesarean section. The routine anaesthetic practice for elective cesarean section at our institute is spinal anaesthesia with a 25G Quincke needle, with the patients in the sitting position. After making them supine, they were randomly assigned to 2 groups: group S (supine) and group K (KCP), and computer-generated randomization was obtained from www.randomizer.org.

Pregnant women with category 1 indications (immediate threat to the life of the mother/fetus), women with contraindications to regional anaesthesia including coagulopathy, local skin infection, uncorrected hypovolemia, bodyweight <50 kg or >100 kg, intrauterine growth restriction, preeclampsia, or multiple gestations, not consenting to regional anaesthesia and those with other significant medical illnesses, were not included in the study. A structured case record form was used to collect data from individual subjects. The independent observer recording data were not involved in individual patient management.

Since the study was done in elective cesarean section, the time taken for patient preparation was not included in the spinal time. Pregnant women recruited for the study were advised standard nil per oral precautions and received aspiration prophylaxis as per the institutional protocol. In the operating room, standard monitoring devices including electrocardiogram (ECG), pulse oximeter, and noninvasive blood pressure cuff were applied. Baseline systolic blood pressure, diastolic blood pressure, mean arterial pressure (MAP), and heart rate (HR) were recorded for all patients. A wide bore intravenous access was secured and all patients received co-loading with 10 mL kg-1 balanced salt solution crystalloid infusion during the performance of the subarachnoid block. The subarachnoid block was performed, with the patient in a sitting position, under standard aseptic precautions using a 25G Quincke needle. All subarachnoid blocks were performed by the same anaesthesiologist who has more than 5 years of experience in anaesthesia practice. Once adequacy of needle position was confirmed by the free flow of CSF, 9 mg of 0.5% hyperbaric bupivacaine and 25 µg fentanyl were injected into the subarachnoid space. The time of drug injection into the subarachnoid space was noted by an independent observer, involved only in data-keeping and not in patient management. The patient was immediately made to lie down after the block placement and positioned as per the allocated group protocol. Subsequently, all the patients were positioned supine with a wedge under the right hip. Supplementary oxygen was administered to all patients via a face mask at 6 L min-1.

Following the subarachnoid block, MAP and HR were recorded every 2 minutes till the first 8 minutes and then every 5 minutes till the end of surgery. Sensory block height was assessed using the loss of sensation to pinprick. The onset of the block at the T6 level was defined as the time interval between spinal injection and loss of sensation to pinprick at the T6 level. Time from spinal injection till attainment of T6 height of sensory block was noted. Surgery was commenced once the T6 dermatomal sensory block was achieved. T6 was chosen to cover for the visceral pain which was well predicted with pinprick testing.^9^ Sensory block was assessed by the independent observer who was blinded to the technique used and entered the operating room 60 seconds after the procedure. The block was assessed till the attainment of maximal sensory block level (MSBL), which was confirmed by a repeat assessment after an interval of 3 minutes. The time at which MSBL was achieved was also recorded. Motor block was assessed using modified Bromage scale (0 = no motor block, 1 = hip flexion blocked, 2 = hip and knee flexion blocked, and 3 = hip, knee, and ankle flexion blocked). Patients who had >20% decrease in heart rate/blood pressure from baseline or an HR of <50 beats per minute were diagnosed as having significant bradycardia/hypotension and received crystalloid boluses and titrated boluses of atropine/ephedrine as per the discretion of the attending anaesthesiologist, respectively. The total requirement of fluids, chronotropic/vasopressor drug boluses in each group were recorded. The respiratory assessment was done by observing the respiratory rate through transthoracic impedance pneumography using an ECG module. The newborn was evaluated using Apgar scores at 1 and 5 minutes.

### Positioning

Supine group (n=22): Pregnant women in this group continued to be positioned supine with a wedge under the right hip.

Knee-chest position group (n=23): Pregnant women in this group were positioned supine with legs drawn up over the abdomen and maintained in that position for 60 seconds.

### Statistical Analysis

A previous series reported that the median (IQR) time of onset was 4 (1.5) minutes which is required for the onset of spinal anaesthesia at T10 level.^2^ In the absence of any previous literature, we assumed that a reduction of onset time around 1.5 minutes will be clinically significant. To detect a difference of 1.5 minutes in the onset time with 90% power, alpha of 0.05, at least 44 patients were required. So, n = 45 patients were randomized in this trial. A total of 51 patients were screened for the study.

All collected data were entered in a Microsoft Excel™ spreadsheet (Microsoft Corp., Redmond, Wash, USA). Data were presented as median and interquartile range (IQR) for continuous variables and as absolute numbers or percentages­ for categorical variables. Non-parametric and categorical variables were compared by Mann–Whitney *U* test, whereas binary variables were compared by chi-square test, and a difference between median with 95% CI was reported. A *P* value less than .05 was considered significant. All statistical analyses were conducted using STATA 12 software for Mac OS (StataCorp. 2011. Stata Statistical Software: Release 12, StataCorp LP, College Station, Tex, USA).

## Results

**Figure 1.**
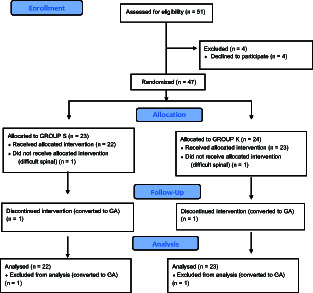
CONSORT flowchart.

Fifty-one patients were initially assessed for inclusion in this study. Four patients were excluded from the study due to inability to obtain consent, 2 patients had a difficult placement of spinal and required conversion to general anaesthesia. So, the data of 45 patients were analyzed. The randomization has been depicted in Figure 1. All patients chosen for the study were full-term pregnancies and had completed 38 weeks of gestation.

**Figure 2.**
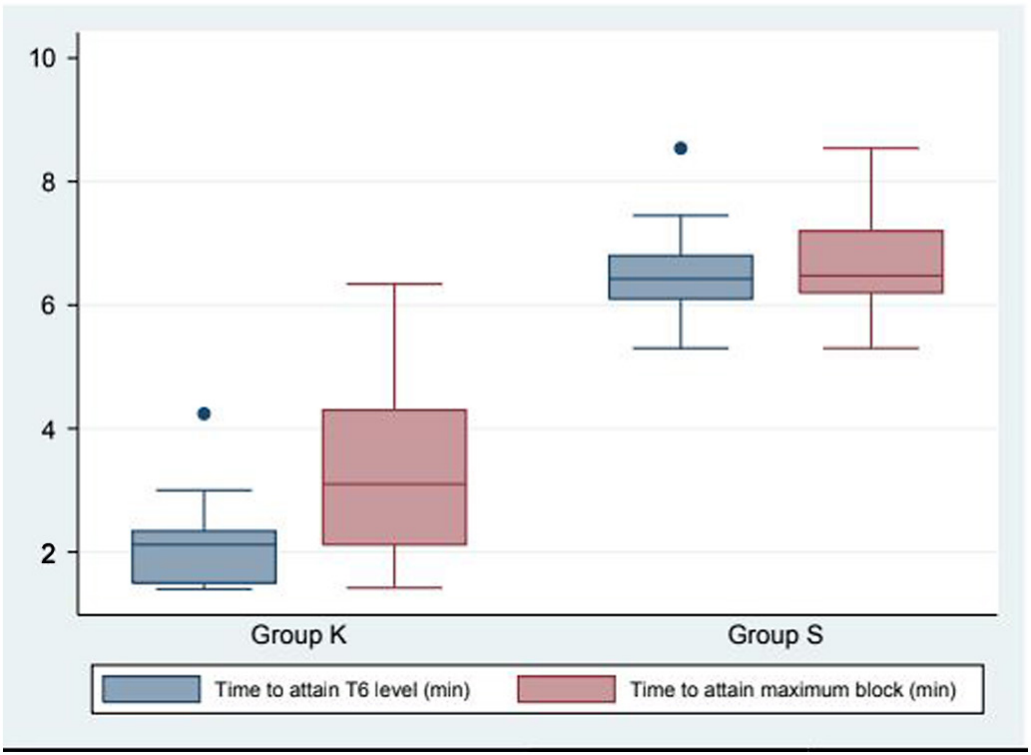
Box–Whisker plot shows the time taken to attain T6 block height in the 2 groups and time to maximum sensory blockade in both the groups.

The baseline demographics of all patients are presented in Table 1. As shown in Figure 2, the median time to attainment of T6 block height was significantly lower in group K compared to group S [median (IQR) 2.1 (1.5-2.3) min vs 6.5 (6.1-6.8) min, the difference in median (95% CI) 4.3 (3.9-4.7) min; *P* < .0001]. The time to maximum sensory blockade in group K was also significantly lower than in group S [median (IQR) 3.1 (2.1-4.3) min vs 6.5 (6.2-7.2) min, the difference in median (95% CI) 3.4 (2.4-4.4) min; *P* < .0001] (Figure 2). However, there was no statistically significant difference in the maximum sensory blockade attained among both groups (*P* = .052). More than 50% of patients in group K had block heights extending above T6. The maximum block height noted in group K was T2, in 2 patients (8.7%). The maximum sensory blockade in group S, however, was T4, which was noticed in only 1 of the 22 patients (4.55%). 

Motor blockade as assessed by modified Bromage scale was significantly higher in group K at 2 minutes (*P* = .0002), 4 minutes (*P* < .0001), and 6 minutes (*P* < .0001); however, no difference was seen at 8 minutes (*P* > .99). At 2 minutes, all the patients in group K showed evidence of motor block. 52.2% of the patients in group K had a Bromage scale of 2 compared to 4.6% in group S. Analysis of the motor block at the fourth minute showed that 60.9% of the patients in group K had complete motor blockade against none in group S. At the sixth minute, all the patients in group K reported complete motor block compared to only 2 patients in group S. The motor block was complete in both the groups at the eighth minute.

**Figure 3.**
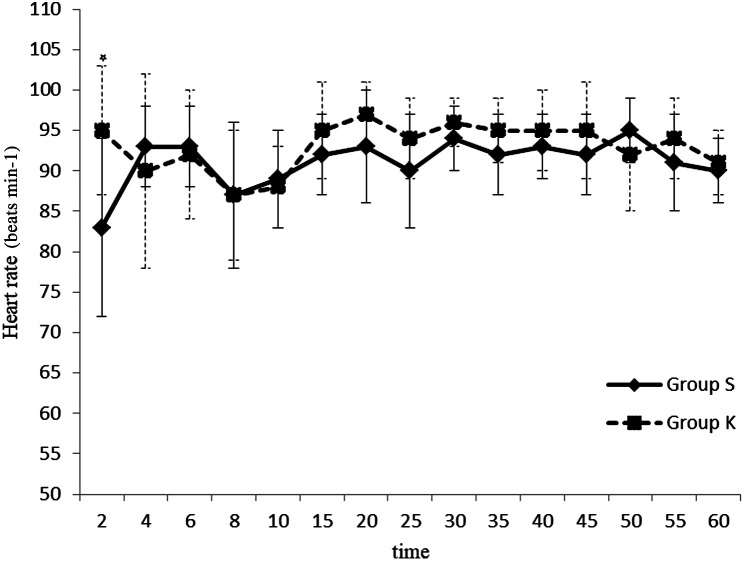
Heart rates in both groups (*P < .0001).

**Figure 4.**
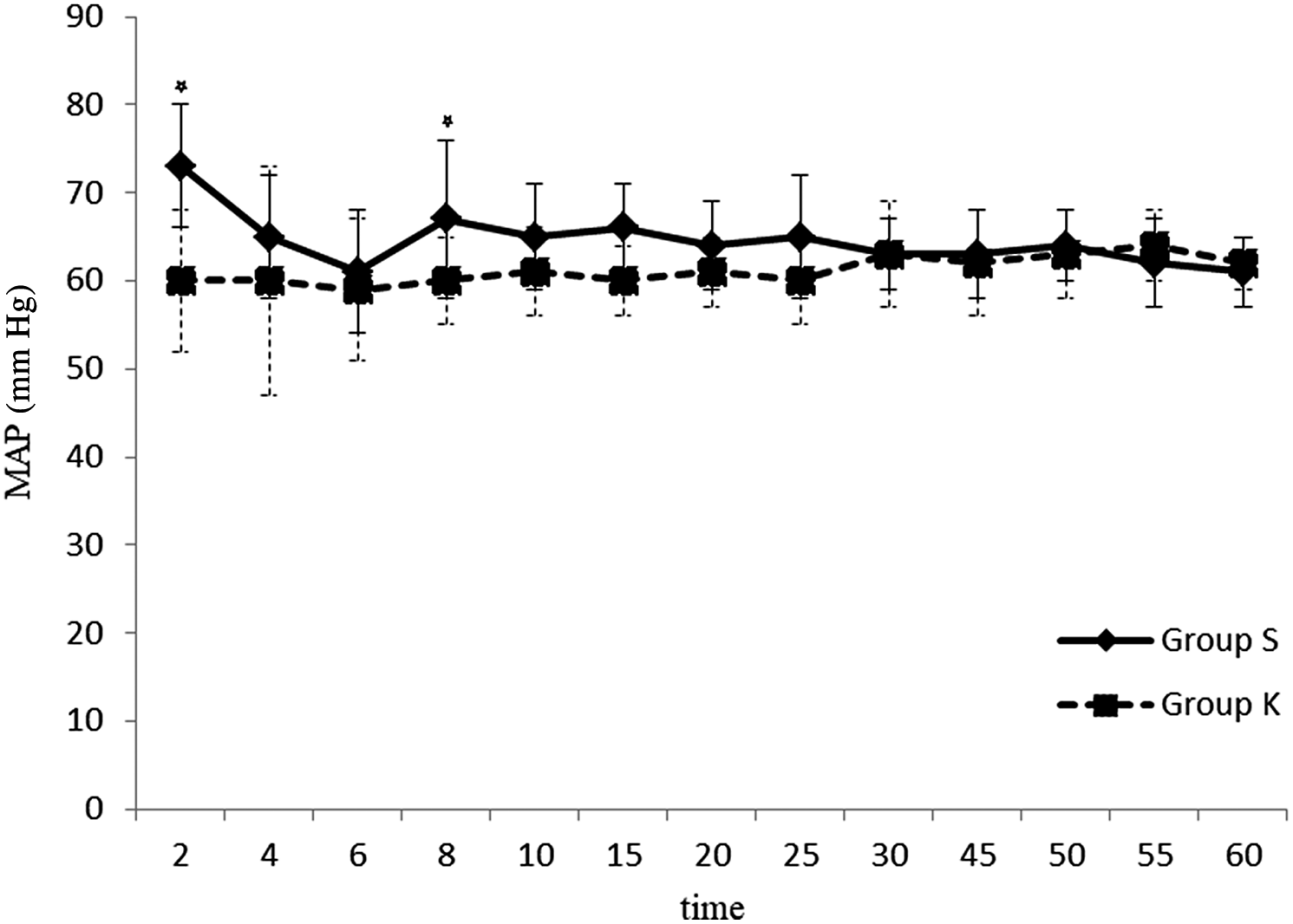
Mean arterial pressure in the 2 groups (*P < .0001).

Heart rates recorded at the second minute showed a statistically significant difference, with parturient in group K having higher HR (94.9 ± 8 beats per minute) than group S (83.4 ± 11 beats per minute) (*P *< .0001). Heart rates at fourth, sixth, and eighth minute did not show any statistically significant difference between the groups (Figure 3). Figure 4 depicts the trends in MAPs in both groups. The MAPs at second and eighth minute following placement of subarachnoid block showed a significant difference between the 2 groups. In the fourth and sixth minute, at all time points beyond, MAPs were comparable in both the groups. No change was observed in the respiratory rate in both groups. 

Nine out of 23 parturients (39.1%) in group K required ephedrine/atropine boluses compared to 3 out of 22 parturients (13.6%) in group S (*P* = .09). Among those who required ephedrine/atropine boluses, there was no statistically significant difference in the dose of ephedrine administered in either of the groups. The total dose of ephedrine and intravenous fluids was calculated in both groups. Mean (SD) ephedrine consumption in group S was 4 (1.73) mg compared to 5.33 (2) mg in group K (*P* = .33). Two patients in group K (8.7%) received atropine boluses while there was no requirement for atropine in group S (*P* = .47)**.** The amount of intravenous fluids administered was also comparable between the 2 groups–1.46 ± 0.37 L in group K vs 1.41 ± 0.37 L in group S (*P* = .65).

The mean Apgar scores were comparable in both groups–[7.44 ± 0.51 vs 7.22 ± 0.71 at 1 minutes (*P* = .23) and 8.63 ± 0.25 vs 8.54 ± 0.36 at 5 minutes (*P* = .33)], in groups K and S, respectively. 

No analgesic supplementation was required in any patient. The mean surgical time from skin incision to delivery of the baby was 8.05 ± 2.43 minutes and from skin incision till skin closure was 20.34 ± 7.32 minutes.

## Discussion

The principal findings of our trial are that a supine with KCP immediately after spinal anaesthesia significantly increased the speed of sensory block and the time to achieve T6 dermatomal level. It was also found to increase motor block up to 6 minutes after spinal anaesthesia. However, MAP was noted lower with KCP up to 8 minutes after spinal anaesthesia.

To the best of our knowledge, the effect of KCP after spinal anaesthesia has not been compared with the supine position in pregnant women. The pregnant state has been associated with decreased local anaesthetic requirements. Various mechanisms have been postulated including anatomical, biochemical, and hormonal changes to explain the lesser local anaesthetic requirement and a faster cephalad drug spread in pregnancy.^10-12^ An increase in the intra-abdominal pressure due to the gravid uterus may facilitate cephalad spread of local anaesthetic by 2 mechanisms–first, by engorgement of epidural venous plexus and second, by inward displacement of soft tissues into the intervertebral foramina, thereby increasing the CSF pressure.^11,13^ Johnston et al.^8^ in a landmark study examined the epidural pressures in 14 laboring women with various position alterations. They found that the supine position with legs raised up had the greatest increase in pressures in the epidural space. The increased epidural pressure would lead to a cephalad surge of CSF. In addition, assuming a KCP may help flatten the exaggerated lumbar lordosis present in such patients and facilitate the free flow of CSF. A shorter time to achieve maximum sensory level is probably due to an increase in epidural pressure from KCP. Kathirgamanathan et al.^6^ in a clinical observational study in 100 category 4 cesarean sections reported a median time of 5.56 minutes to the onset of adequate surgical anaesthesia after subarachnoid block. The adequacy of the block was, however, assessed by the anaesthetist’s opinion of readiness for surgery rather than a predefined objective parameter.

Thus, in our study population, using the KCP following placement of subarachnoid block was significantly beneficial in attaining the desired surgical anaesthesia faster. However, more cephalad spread of local anaesthetic carries a higher risk of hypotension and may increase the fluid and vasopressor requirement. Though we have not found any difference in fluid and vasopressor requirements, blood pressure was lower in the KCP group up to 8 minutes after spinal anaesthesia. The HR values at the second minute following subarachnoid block were higher in KCP, possibly due to a compensatory sympathetic response to lower blood pressures in this group. This faster attainment of block height, while desirable in practice, carries with it an inherent risk of causing greater hemodynamic instability. Zhang et al.^14^ reported the role of spinal block ascension rate on intraoperative hypotension. Therefore, while performing such a maneuver, the anaesthesiologist should be ready to deal with lower blood pressure readings at least in the initial phase following placement of the subarachnoid block. On the other hand, a study by Ozkan et al.^15^ found a poor correlation between the increased intra-abdominal pressure of pregnancy and subarachnoid block height, thereby questioning the influence of a raised intra-abdominal pressure on cephalad spread of CSF. However, they did not assess the effect of change in maternal posture on intra-abdominal pressure.

We specifically chose a lower dermatomal target for spinal anaesthesia as our clinical experience suggests that cesarean section is usually performed uneventfully after attainment of T6 dermatomal level. We used 9 mg hyperbaric bupivacaine as the mean height of our patients and in the Asian population, in general, is lower than the western population. 

The most important clinical application of our study in patients undergoing category 1 cesarean section as quick delivery of the fetus is of utmost importance in that scenario. However, the positioning of the patient requires cooperation and adequate training of the operating room staff to save time, which is a key factor for use of neuraxial anaesthesia in category 1 sections. The actual time taken to perform spinal anaesthesia is often equivalent to the performance of general anaesthesia but the time needed to attain adequate surgical anaesthesia after an optimally placed spinal block is unpredictable and widely variable.^6^ On the other hand, following induction of general anaesthesia, the operating obstetrician can commence surgery following secure placement of the endotracheal tube. In a survey performed in the United Kingdom, general anaesthesia continued to be used widely for category 1 cesarean sections.^3^ Further clinical studies are required to validate KCP to hasten the block quality in these patients. 

In this context, the study by Kinsella et al.^7^ described a novel rapid sequence spinal technique to attain a satisfactory spinal block in about 4 minutes. However, the study predominantly shortened the duration of spinal induction by using lesser aseptic precautions, using separate staff for monitoring and cannulation, and avoiding local anaesthetic infiltration. Thus, there are concerns about exposing the parturient to the lesser sterile techniques and the possible risks of sepsis with this technique. While training in the use of a rapid sequence spinal technique has been offered to anaesthesiologists in England, many are still reluctant to use it in real-life practice.^3^ The KCP maneuver in our study halved the time to adequate surgical anaesthesia in comparison to the study by Kinsella et al.^7^ while avoiding the risks associated with a less sterile technique.

Our study has few limitations. First, we did not measure the changes in epidural pressure following KCP. This could have provided us with more objective information regarding the magnitude of role played by pressure changes in the cephalad spread of the local anaesthetic. Second, we used lower doses of bupivacaine in our study as women of the Asian population are generally shorter in stature in comparison to their western counterparts. Our experience suggests that such a lower dose of bupivacaine works well in getting the desired level of surgical anaesthesia in our population with minimal hemodynamic perturbations. However, the results of this study cannot be generalized as dose ranges may vary depending upon the build of the parturient.

## Conclusion

In conclusion, the current study provides enough evidence that the onset of adequate surgical anaesthesia for the cesarean section can be hastened by placing the patient in KCP for a minute immediately after the administration of the subarachnoid block. The hemodynamic effects of this technique were safe in our patient population though the anaesthesiologist should be well prepared to encounter lower blood pressure readings in the initial minutes following placement of the block. 

## Figures and Tables

**Table 1.** Demographic Parameters of the Patients in Both the Groups

Variable	Group S (n = 22)	Group K (n = 23)	*P*
Age (years)	29 [26-34]	30 [27-32]	.93
Weight (kg)	67 [59-72]	65 [60-76]	.89
Height (cm)	160 [156-165]	160 [156-164]	.90
BMI (kg/m^2^)	26.5 [22.5-28.1]	25.4 [23.1-29.4]	.95
Gravida Primi Multi	8 14	10 13	.86
Singleton Twin	20 2	22 1	.97

Data represented as median (IQR) or proportion.
